# MIRN: A multi-interest retrieval network with sequence-to-interest EM routing

**DOI:** 10.1371/journal.pone.0281275

**Published:** 2023-02-02

**Authors:** Xiliang Zhang, Jin Liu, Siwei Chang, Peizhu Gong, Zhongdai Wu, Bing Han

**Affiliations:** 1 College of Information Engineering, Shanghai Maritime University, Shanghai, China; 2 Shanghai Ship and Shipping Research Institute, Shanghai, China; 3 COSCO Shipping Technology Co., LTD, Shanghai, China; Zhejiang University of Technology, CHINA

## Abstract

Vector-based retrieval have been widely adopted to process online users’ diverse interests for recommendations. However, most of them utilize a single vector to represent user multiple interests (UMI), inevitably impairing the accuracy and diversity of item retrieval. In addition, existing work often does not take into account the scale and speed of the model, and high-dimensional user representation vectors need high computation cost, leading to inefficient item retrieval. In this paper, we propose a novel lightweight multi-interest retrieval network (MIRN) by incorporating sequence-to-interest Expectation Maximization (EM) routing to deal with users’ multiple interests. By leveraging representation ability of the Capsule network, we design a multi-interest representation learning module that clusters multiple Capsule vectors from the user’s behavior sequence to represent each of their interests respectively. In addition, we introduce a composite capsule clustering strategy for the Capsule network framework to reduce the scale of the network model. Furthermore, a Capsule-aware module incorporating an attention mechanism has been developed to guide model training by adaptively learning multiple Capsule vectors of user representations. The experimental results demonstrate MIRN outperforms the state-of-the-art approaches for item retrieval and gains significant improvements in terms of metric evaluations.

## 1 Introduction

**Fig 1:**
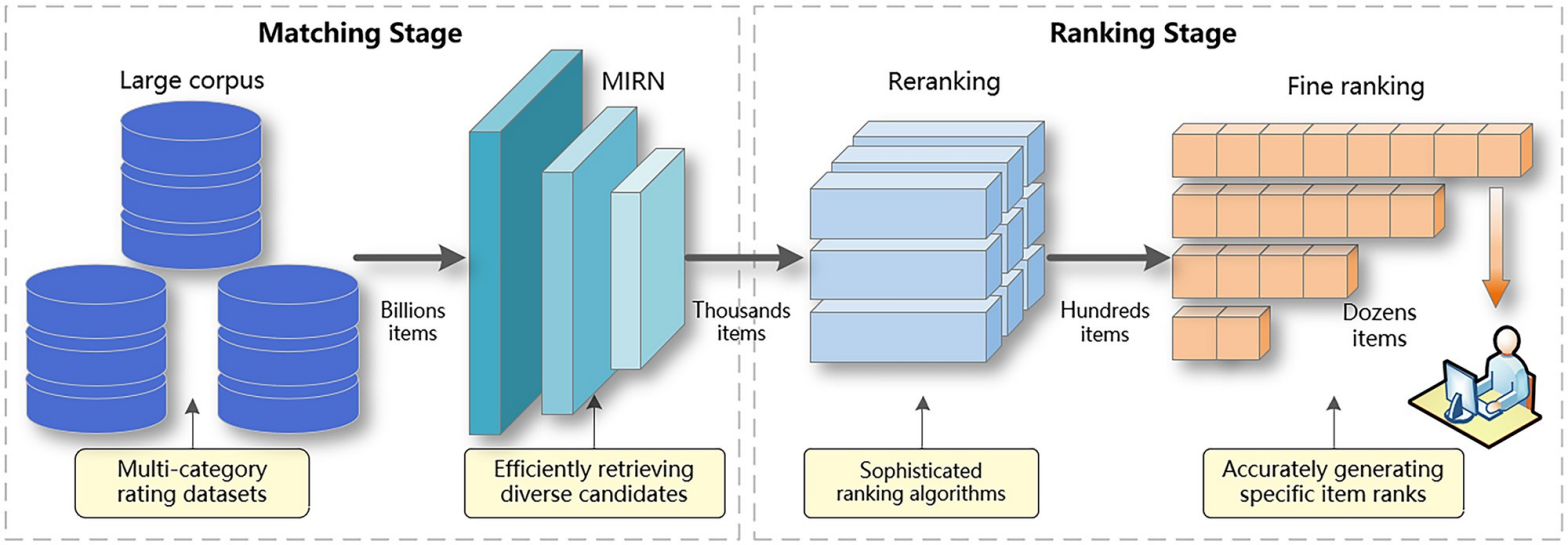
An illustrative diagram shows how to use MIRN in an industrial recommendation system. MIRN focuses on the matching stage, which aims to retrieve user-interested items efficiently. Note that the matching stage is concerned with retrieving good items, rather than specific item ranks.

Recommendation systems are an effective way to alleviate information overload and suggest relevant options to end users to satisfy their individual needs and interests, especially in many user-oriented online services, such as e-commerce (Amazon, Taobao) and social media (Facebook, Instagram) sites [1]. Recommendation systems usually consist of two stages, namely the matching stage and the ranking stage. Fig 1 shows an illustrative diagram of this process. Here, the purpose of matching stage is to efficiently retrieve a subset of items from the entire corpus that are relevant to user’s interests. That is, the massive multi-category rating dataset stored in the corpus is retrieved by our model to generate thousands of candidate items related to users’ multiple interests during the matching stage. In the ranking stage, the retrieved items not only need to be refined by accurate recommendation algorithms and scoring algorithms but also need to be adjusted in conjunction with specific business rules to produce the final items to be recommended. As the initial phase of the entire recommendation process, the performance of the retrieval model determines the upper limit of the final recommendation. Therefore, it is critical to model user interests and find user representations for matching stage in order to retrieve items that satisfy user interests.

Recently, vectorization-based retrieval methods have been widely adopted, such as DSSM [2], Deep FM [3], YouTube DNN [4], etc. The basic idea is to embed users and items into a potential vector space and utilize the inner product of vectors to represent the preferences between users and items. Although these methods have made some progress in the matching stage, existing recommendation models still have bottlenecks in learning multiple interest embeddings from user behavior sequences. Besides, knowledge graph-based approaches, such as Deep Walk [5], EGES [6], and Graph SAGE [7], are used to investigate recommendation diversity. The fundamental idea is to construct a knowledge graph utilizing users’ behavioral data and then develop a function that performs an aggregated representation of surrounding vertices to provide an embedding vector of target items. While the knowledge graph-based approach makes a small contribution to solving the UMI problem, requirements for huge computational cost and storage space make it difficult to deploy on devices with limited resources. On the one hand, the size of the model and the algorithm’s inference speed are the pressing issues that we need to address in our deployment. On the other hand, the amount of user-item interactions amassed in online service platforms grows over time, and user behavior sequences keep becoming larger, so extracting merely a single embedding vector from them is no longer feasible. In fact, the matching stage should take more responsibility for diversity since it is more concerned with the coverage of user-interest items, which directly determines the upper limit of recommendation effectiveness. The diversity of recommenders needs to be ensured in the matching stage first. Otherwise, the homogeneity of candidate items generated by the matching stage will inevitably lead to a lack of diversity in the final recommendations.

In this paper, to overcome above limitations, we focus on the problem of modeling different interests of users, which is crucial in real-world recommender systems. We propose a lightweight Multi-Interest Retrieval Network (MIRN) with Sequence-to-Interest Expectation Maximization routing (EM) to reflect user representations with different interests. Specifically, we design a multi-interest representation learning module that uses Sequence-to-Interest Expectation Maximization routing (S2I-EM) to adaptively cluster users’ historical behaviors into user representations. For a particular user, MIRN outputs multiple representation vectors, and each of them represents one interest tendency of the user, respectively. Ultimately these representation vectors can be used in the matching stage to retrieve user-interest related items from a large corpus. We conduct extensive experiments on two public datasets. The experimental results confirm that MIRN exhibits excellent performance compared to current state-of-the-art models. To summarize, the main contributions of this work are as follows:

- To obtain UMI from behavior sequences, we designed a multi-interest representation learning module incorporating S2I-EM routing, which adaptively clusters users’ behavior sequences into multiple representation vectors. This work lays a good foundation for the successful application of EM routing algorithms in the matching stage.
- In order to reduce the size of our model and to be able to deploy it smoothly in real scenarios, we designed a composite capsule clustering strategy and applied multiple ways to speed up the computation of algorithm iterations. This is the first study to apply a model that integrates three characteristics (accuracy, diversity, and complexity) for item retrieval.
- Extensive experiments conducted on two real-world datasets demonstrate that MIRN achieves significant better performance consistently.

## 2 Related work

Early work on item retrieval typically used collaborative filtering (CF) to model user preferences based on their interaction history [8, 9]. Model-based collaborative filtering approaches [10–15] are an increasingly common work line. These approaches project users and items into a shared vector space and estimate a user’s preference for an item by measuring the similarity between the user and the items in her/his interaction history using a pre-computed item-to-item similarity matrix. In practice, however, the number of users is frequently much greater than the number of items, resulting in a significant overhead in maintaining and storing these similarity matrices. Furthermore, a user’s historical data is frequently minimal, and the accuracy of finding similar users who have only made a few transactions or clicks is low.

In the last few years, tree-based approaches have been extensively studied in the item matching stage [16–18], especially in capturing the diversity of user interests. Tree-based retrieval approaches [19–23] use a tree structure as an index and attach each item in the corpus to a leaf node by clustering [23] or joint learning [22]. Specifically, a node in the tree represents an embedding vector, and the model outputs a probability value corresponding to that vector by computing it. The probability value is used to describe the user’s interest in a particular node, and the whole tree represents the hierarchical information about the user’s interest (diversity of user interests). In addition, although the tree-based retrieval algorithm allows the model to learn more information about the interaction between users and candidate items (improve the accuracy of item retrieval), the complexity of the structure and algorithm limits it from wide application in industry. Therefore, tree-based retrieval algorithms have greater potential for improvement both theoretically and engineering-wise.

Since then, a number of vector-based approaches for diversifying retrieval results have been proposed. Embedding approaches have had a major impact in both academia and industry. Covington et al. [4] mapped users and items into a low-dimensional dense vector in a YouTube video recommendation system, and then performed item retrieval by an efficient retrieval method. In the deep-attention network (DAN) [24], the self-attention mechanism guides the model to appropriately filter feature vectors to reduce the interference of redundant information. Huang et al. [25] developed a vector embedding-based retrieval framework and applied it to an inverted index-based search system (Facebook). This work is a successful practice of vector embedding-based retrieval algorithms deployed to large search systems. Nie et al. [26] proposed a novel cross-domain learning network (CLN) for 2D image-based 3D shape retrieval task. Xinyang, Yi et al. [3] proposed a neural deep retrieval (NDR) algorithm based on a two-tower network architecture for retrieving personalized suggestions from a corpus of tens of millions of videos (YouTube). The core idea is to encode a wide variety of item feature vectors using a network called item tower. An advantage of these vector embedding-based retrieval methods is that quantization-based indexing [27] techniques and hierarchical graph indexing [28] techniques can be employed to speed up their retrieval efficiency.

Recently, several researchers have attempted to use graph neural networks [29–33] for item retrieval [34, 35]. Hamilton et al. presented GraphSAGE [7], a new graph learning model that learns the embedding representation of the target node by aggregating the feature information of the node’s neighbors, i.e., to improve the user-side representation and accuracy in the matching stage. Ying et al. [36] created PinSAGE, an efficient graph convolution approach that enriches item-side representation by generating item-based knowledge graphs. Besides, to improve the diversity and robustness of item retrieval, the model is trained with increasingly difficult samples. While graph-based retrieval approaches can take into consideration the diversity of user interests, the complexity of knowledge graph construction, as well as the higher storage capacity and processing capability requirements, are the most prominent challenges for deployment in real industry applications.

## 3 Methodology

### 3.1 Problem formalization

In this article’s recommendation scenario, we let $U=\{u_{1},u_{2},\ldots ,u_{\left|U\right|}\}$ be the user set and I be the complete items pool, where |*u*| is the total number of unique users. Each user u∈U can be associated with an item set $I_{u}\in I$, i.e., $I_{u}$ stores all items interacted by user *u* (also called user behavior). The goal of our framework is to retrieve a subset of items from a million-scale items pool $I-I_{u}$ for each user u∈U such that the subset contains only a few thousand items and each item is relevant to the user’s interests. In addition, the basic information (like user gender and age) of user *u* can be represented by $B_{u}$, and $F_{i}$ is used to represent the characteristics of the target item, such as item id and category id.

The core task of MIRN is to learn a function that maps raw features into user representations, which can be expressed as

$$\begin{array}{cc} & M_{u}=f_{user}\left(B_{u},I_{u}\right), \end{array}$$

where the $M_{u}=\left\{{\vec{m}}_{u}^{1},{\vec{m}}_{u}^{2},\ldots {\vec{m}}_{u}^{K}\right\}\in R^{d\times K}$ denotes the representation vectors of user *u*. *d* is the dimensionality, and K denotes the number of representation vectors. In this paper, we use a dynamic strategy to replace the fixed parameter K. The details are described in Eq (16). Besides, the representation vector of target item *i* is obtained by an embedding function as

$$\begin{array}{cc} & {\vec{v}}_{i}=f_{item}\left(F_{i}\right), \end{array}$$

where ${\vec{v}}_{i}\in R^{d\times 1}$ denotes the representation vectors of item *i*. Now a similarity score can be computed based on the user representation vector and the item representation vector to filter out the top *N* items related to the interests of each user *u* from the item candidate pool. The scoring function is as follows:

$$\begin{array}{cc} & f_{score}\left(M_{u},{\vec{v}}_{i}\right)=\text{max}\left\{{\vec{v}}_{i}^{⊤}{\vec{m}}_{u}^{k}\right\},1\leq k\leq K, \end{array}$$

where *N* is the final number of candidates, which is set in advance based on the whole system. The main notations we used are listed in Table 1.

**Table 1:** Glossary.

Symbol	Description
U	the set of users
I	the items pool
$I_{u}$	the item set interacted by user *u*
$B_{u}$	basic information of user *u*
$F_{i}$	the characteristics of target item *i*
$M_{u}$	the representation of user *u*
${\vec{v}}_{i}$	the representation of item *i*
P	the lower layer behavioral Capsules
Q	the upper layer interest Capsules
K	the expected number of interests
$R_{ij}$	the connection probability
$V_{ij}$	the vote matrix
*N*	the number of candidates

### 3.2 Embedding layer

**Fig 2:**
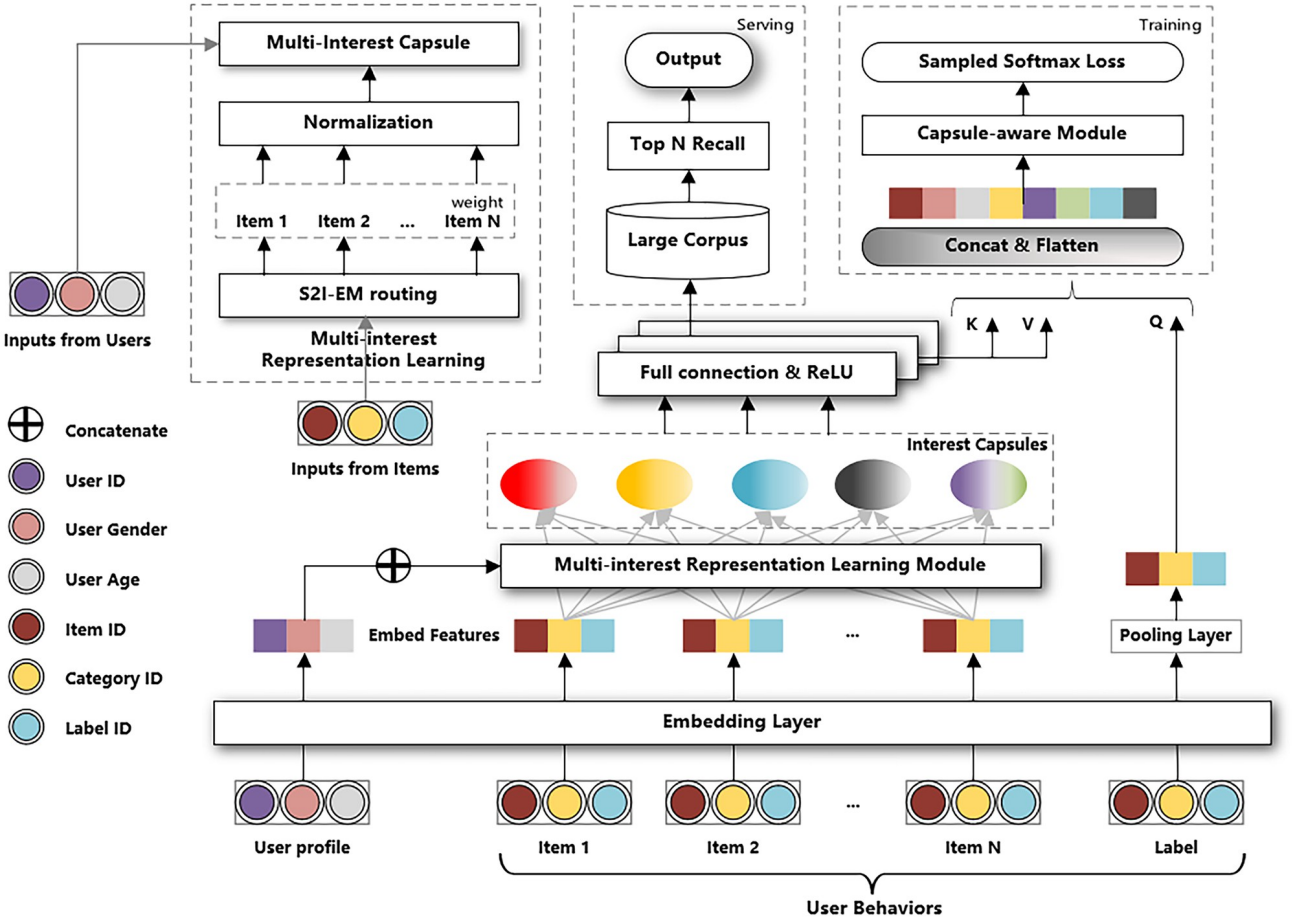
Network structure of MIRN. MIRN takes a sequence of user profile features and user behaviors as input and outputs multiple user representation vectors for item retrieval. User behaviors and other user features (gender, age, etc.) are fed to the multi-interest representation learning module through the corresponding operations (feature embedding and average pooling) of the embedding module. Then, the interest Capsules generated by the multi-interest representation learning module are stitched with the user’s other feature embeddings separately, and multiple user representation vectors are obtained through several Full connection & ReLU layers. Finally, multiple user representation vectors are used in parallel for Top N item retrieval. The Capsule-aware module is mainly used to guide the training of the model.

As shown in Fig 2, the input of MIRN contains three parts, user basic information $B_{u}$, user behavior sequence $I_{u}$, and label item $F_{i}$. The label item is split from user behaviors and belongs to a recent real purchase made by a user. Due to the large size of the dataset, the dimensionality of each feature is high, especially some categorical id features, so it is difficult to operate with one-hot encoding and the similarity between features is difficult to capture. To reduce the number of parameters in the computation process, we adopt the word2vec [37] embedding technique to embed high-dimensional features into low-dimensional dense vectors, and each feature of this dense vector can be considered to have practical value. Specifically, for users containing features such as age and gender from user basic information $B_{u}$ are encoded into user feature embeddings ${\vec{b}}_{u}$, while item ids along with other categorical ids from $F_{i}$ are encoded to form the label item embedding ${\vec{v}}_{i}$ after averaging the pooling layer. And the items interacted by users from $I_{u}$, corresponding item embeddings are obtained to form the user behavior embedding.

### 3.3 Multi-interest representation learning

The multi-interest representation learning module is developed to learn multiple representation vectors from the user’s behavior sequence, and finally to form the corresponding interest Capsule of the user.

#### 3.3.1 Expectation maximization routing

We begin with a brief review of the important basics about Expectation Maximization (EM) routing, an iterative algorithm that can also be considered as a new form of neural units represented by matrices. The original EM routing was designed for image processing by computing pixel values to update background images, cannot being applied for recommendation task. However, the powerful feature capture and attribute processing capabilities exhibited by EM routing at the fine-grained level provide us an important insight that we may use it to handle category properties with strong entity representation in recommendation tasks.

Another key basis is the Gaussian Mixture Model (GMM), a clustering method utilized in EM routing. The advantages of the GMM can be summarized as follows: on the one hand, it can handle nonlinear decision boundaries and produce good clustering results. On the other hand, it can cluster data based on its distribution and interpret the clustering results more accurately. In this paper, we suppose that the user historical behavior data can be separated into K categories and that they all follow a Gaussian distribution, the probability density function of GMM may be stated as:

$$\begin{array}{ccc} & P\left(x\right)=\sum_{j=1}^{K}P\left(j\right)P\left(x|j\right) & \\ & =\sum_{j=1}^{K}\pi_{j}N\left(x;\mu_{j};\Sigma_{j}\right) & \end{array}$$ (1)

where *j* represents the category, P(j) denotes the probability of each category being chosen, usually written as *π*_*j*_. The probability distribution P(x|j) for each category/interest can be expressed as:

$$\begin{array}{cc} & N\left(x;\mu_{j};\Sigma_{j}\right)=\frac{1}{\sqrt{{\left(2\pi \right)}^{d}\left|\Sigma_{j}\right|}}exp^{\left(-\frac{1}{2}{\left(x-\mu_{j}\right)}^{⊤}\Sigma_{j}^{-1}\left(x-\mu_{j}\right)\right)} \end{array}$$ (2)

where *d* represents the dimensionality of input vector/matrix *x*. *μ*_*j*_, Σ_*j*_ are the mean (vector) and covariance (matrix) of the GMM distribution, respectively.

Intuitively, the values of three parameters *π*_*j*_, *μ*_*j*_ and Σ_*j*_ need to be determined during the UMI modeling. There is a deduction based on the Wiener-khinchin law of large numbers, for a Gaussian distribution, the results of default maximum likelihood estimation are equal to the second-order moment estimation in a large enough amount of data. We arrive at a simplified version of the derivation process based on Bayesian definition and the formula is as follows:

$$\begin{array}{ccc} & P\left(j|x\right)=\frac{P(x|j)P(j)}{P(x)} & \\ & =\frac{\pi_{j}N\left(x;\mu_{j};\Sigma_{j}\right)}{\sum_{j=1}^{K}\pi_{j}N\left(x;\mu_{j};\Sigma_{j}\right)} & \end{array}$$ (3)

The mean *μ* can be calculated as:

$$\begin{array}{ccc} & \mu_{j}=\int P\left(x|j\right)=E[\frac{P(j|x)}{P(j)}x] & \\ & =\frac{1}{n}\sum_{i=1}^{n}\frac{P(j|x_{i})}{P(j)}x_{i} & \\ & =\frac{1}{\pi_{j}n}\sum_{i=1}^{n}P\left(j|x_{i}\right)x_{i}c & \end{array}$$ (4)

Similarly, the covariance matrix Σ and the parameter *π*_*j*_ are calculated as follows:

$$\begin{array}{ccc} & \Sigma_{j}=\frac{1}{\pi_{j}n}\sum_{i=1}^{n}P\left(j|x_{i}\right)x_{i}{\left(x_{i}-\mu_{j}\right)}^{⊤}\left(x_{i}-\mu_{j}\right) & \end{array}$$ (5)

$$\begin{array}{cc} & \pi_{j}=\frac{1}{n}\sum_{i=1}^{n}P\left(j|x_{i}\right) \end{array}$$ (6)

The above parameters vary in the S2I-EM routing algorithm inexorably, regulating the quality of user interest diversity. Obtaining their maximum likelihood estimates would be ideal, however they do not have a close form analytical solution. Therefore, we propose the S2I-EM routing algorithm for solving the above problem.

#### 3.3.2 Sequence to interest expectation maximization routing

There are two layers in our routing framework, the lower layer is the sequence of user’s behaviors (behavioral Capsules) and the upper layer is the Capsules obtained by algorithmic clustering, which are user’s interest Capsules. In this paper, $P_{i},\left(1\leq i\leq n\right)$ is used to denote lower layer behavioral Capsules, and *n* is the number of users’ behavioral Capsules. We let $Q_{j},\left(1\leq j\leq K\right)$ denote the upper Capsules, where K is the number of interest Capsules/categories. The goal of S2I-EM routing is to compute the values of upper Capsules in an iterative manner once the values of lower Capsules are known. The specific details will be discussed in the following four ways.

1) ***Covariance matrix replacement with a diagonal matrix***. Without loss of generality, the covariance matrix Σ_*j*_ is defined as a diagonal matrix, i.e., $\Sigma_{j}=diag\sigma_{j}^{2}$, where $\sigma_{j}^{2}$ is the variance vector of category *j*. Therefore, the variance vector is used to describe the covariance matrix mainly for the following considerations. On the one hand, we can avoid solving the inverse of a matrix as well as the determinant, which can greatly reduce memory consumption and computation, improving efficiency of algorithm. On the other hand, *σ*_*j*_ is a standard deviation vector, and $\sigma_{j}^{l}$ denotes the *l*-*th* component of this standard deviation vector, which makes each component of input vector/matrix *x* decoupled and remains independent. Based on the above analysis, the simplification of Eq (2) can be obtained as:

$$\begin{array}{cc} & N\left(x;\mu_{j};\Sigma_{j}\right)=\Pi_{l=1}^{d}\frac{1}{\sqrt{2\pi }\sigma_{j}^{l}}exp^{\left(-\frac{1}{2{\left(\sigma_{j}^{l}\right)}^{2}}{\left(x_{i}^{l}-\mu_{j}^{l}\right)}^{2}\right)} \end{array}$$ (7)

where $\mu_{j}^{l}$ denotes the *l*-*th* component of mean vector of interest *j*. Similarly, the solution expression of Σ_*j*_ is converted to the expression of $\sigma_{j}^{2}$ by the following:

$$\begin{array}{cc} & \sigma_{j}^{2}=\frac{1}{\Sigma_{i=1}^{n}P\left(j|x_{i}\right)}\sum_{i=1}^{n}P\left(j|x_{i}\right){\left(x_{i}-\mu_{j}\right)}^{2} \end{array}$$ (8)

As a result, we may construct a local representation of the S2I-EM routing, as shown in Algorithm 1.

**Algorithm 1**: Local version of *S*2*I* EM Routing

**1** **Procedure S2I EM ROUTING**

**2** ∀_*i*_ ∈ Ω_*i*_, *j* ∈ Ω_*j*_: **Initialize** $R_{ij}=\frac{1}{\left|\Omega_{j}\right|}$

**3** **for** *iter=1,…,r* **do**

**4**  for all behavior capsule *i*: $p_{ij}\leftarrow N\left(P_{i};\mu_{j};\sigma_{j}^{2}\right)$

**5**  for all behavior capsule *i*: $R_{ij}\leftarrow \frac{\pi_{j}p_{ij}}{\sum_{j=1}^{k}\pi_{j}p_{ij}}$, $r_{ij}\leftarrow \frac{R_{ij}}{\sum_{i=1}^{n}R_{ij}}$

**6**  for all interest capsule *j*: $Q_{j}\leftarrow \sum_{i=1}^{n}r_{ij}P_{i}$

**7**  for all interest capsule *j*: $\sigma_{j}^{2}\leftarrow \sum_{i=1}^{n}r_{ij}{\left(P_{i}-Q_{j}\right)}^{2}$

**8**  for all interest capsule *j*: $\pi_{j}\leftarrow \frac{1}{n}\sum_{i=1}^{n}R_{ij}$

**9** **end**

**10** **return** $\left\{Q_{j},\pi_{j}\right\}$


$$\begin{array}{cc} & p_{ij}=N\left(P_{i};\mu_{j};\sigma_{j}^{2}\right) \end{array}$$ (9)

$$\begin{array}{cc} & R_{ij}=p\left(j|x_{i}\right) \end{array}$$ (10)

In this paper, *p*_*ij*_ and $R_{ij}$ are two simple notations for Eqs (9) and (10), respectively. And $R_{ij}$ is the connection probability between behavioral and interest Capsules, which we initialize to a uniform distribution. The calculation of upper interest Capsule $Q_{j}$ will be discussed in Eq (15). The connection probability $R_{ij}$ is used to quantify the correlation between the behavior Capsules and the interest Capsules. For example, if cluster B (lower-level behavioral Capsule) and cluster A (upper-level interest Capsule) are independent of each other, then the connection probability between them is 0. The high correlation between them corresponds to a high connection probability value. Furthermore, the connection probability of a Capsule is governed by the activation probability, i.e., the Capsule with a zero activation probability value also has a zero connection probability.

2) ***A composite capsule clustering strategy***. It is important to select K behavioral Capsules at random to represent the mean value (centroid) of each large interest cluster in the GMM clustering procedure, and then update this mean value in iterations. However, the cost of iterative updates to the GMM is enormous, which not only increases the computing overhead of our model but also severely affects the convergence speed of the algorithm. In addition, large-size models are difficult to deploy in real scenarios where storage space is limited. To ligthen the architecture of our model and retain the advantages of GMM clustering, we designed a composite capsule clustering strategy. Specifically, we first apply a lightweight clustering algorithm, namely *K*-*means*, to accomplish coarse-grained clustering of user behavioral Capsules and generate K data clusters. The GMM algorithm is then utilized to complete a fine-grained partitioning of the K data clusters, with the cluster results/centroids acquired from *K*-*means* serving as one of the inputs to the GMM algorithm. The following are some of the benefits of this strategy: 1) Since the cluster centroids supplied by *K*-*means* are identical to the mean *μ* discovered by GMM, the computation time of mean *μ* in GMM can be considerably reduced. 2) The composite clustering technique minimizes the number of GMM iterations, reduces the size of the model, and speeds up the algorithm’s convergence. Note that we also pre-set a value for the stop iteration operation, which can be used to force termination if the algorithm does not converge for a long time.

3) ***Information entropy describes activation value***. Similar to traditional neural networks, we use a scalar *a*_*j*_ to measure the salience of Capsule features in the information propagation process of S2I-EM routing, where we call *a*_*j*_ the activation value of a Capsule. Then we ask: *how to obtain the activation value?* A promising solution is as follows:

We can cluster users’ interests by using GMM and obtain a probability distribution *p*(*x*|*j*), which can describe a user-specific interest Capsule/category. The salience of Capsules can be indirectly measured by the degree of solidarity, so we introduce information entropy in S2I-EM routing to quantify the activation value. Specifically, the salience of a Capsule can be represented by the information entropy, and the higher information entropy (close to uniform distribution), the smaller activation value. On the contrary, the lower information entropy (the closer to the Gaussian distribution), the higher activation value of the category. The information entropy $S_{j}$ in our S2I-EM routing is defined in the following way:

$$\begin{array}{cc} & S_{j}=-\sum_{i=1}^{n}r_{ij}\;\text{ln}\;p_{ij}=\frac{d}{2}+\left(\sum_{l=1}^{d}\;\text{ln}\;\sigma_{j}^{l}+\frac{d}{2}\;\text{ln}\;\left(2\pi \right)\right)\sum_{i=1}^{n}r_{ij} \end{array}$$ (11)

We believe that the high information entropy of a category group indicates that the current category group is still chaotic. Take for example, there is still interest belonging to category B clustered in category A, making the Capsules of category A clusters hold less weight in the voting process. Conversely, when the information entropy is small, it means that the cluster is close to convergence and can contribute to the upper-level Capsule, hence the corresponding activation value should be larger.

Here we have supplemented the derivation of Eq (11). Information entropy can be used to measure the degree of confusion of a category, which in turn can describe the activation value of the category. Firstly, we give the formula for calculating information entropy as:

$$\begin{array}{ccc} & S_{j}=-\int p\left(x|j\right)\;\text{ln}\;p\left(x|j\right)dx & \\ & =-\frac{1}{p(j)}\int p\left(j|x\right)p\left(x\right)\;\text{ln}\;p\left(x|j\right)dx & \\ & =-\frac{1}{p(j)}E\left[p\left(j|x\right)\;\text{ln}\;p\left(x|j\right)\right]\\ & =-\frac{1}{n\pi_{j}}\sum_{i=1}^{n}R_{ij}\;\text{ln}\;p_{ij} & \\ & =-\frac{1}{\sum_{i=1}^{n}R_{ij}}\sum_{i=1}^{n}R_{ij}\;\text{ln}\;p_{ij} & \\ & =-\sum_{i=1}^{n}R_{ij}\;\text{ln}\;p_{ij} & \end{array}$$ (12)

Note that, one of the essential reasons why we use the above procedure to calculate the information entropy instead of integrating it directly is that the latter calculates the theoretical result and the former obtains the actual result for this batch of data.

Next, we work on the derivation of the information entropy in S2I-EM routing. Here, it is assumed that *p*_*ij*_ corresponds to an independent normal distribution of *d* elements with the following equation:

$$\begin{array}{ccc} & S_{j}^{l}=-\sum_{i=1}^{n}r_{ij}\;\text{ln}\;p_{ij}^{l} & \\ & =-\sum_{i=1}^{n}r_{ij}\;\text{ln}\;[\frac{1}{\sqrt{2\pi }\sigma_{j}^{l}}exp^{\left(-\frac{{\left(x_{i}^{l}-\mu_{j}^{l}\right)}^{2}}{2{\left(\sigma_{j}^{l}\right)}^{2}}\right)}] & \\ & =\left(\frac{1}{2}\;\text{ln}\;2\pi +\;\text{ln}\;\sigma_{j}^{l}\right)\sum_{i=1}^{n}r_{ij}+\frac{\sum_{i=1}^{n}r_{ij}{\left(x_{i}^{l}-\mu_{j}^{l}\right)}^{2}}{2{\left(\sigma_{j}^{l}\right)}^{2}} & \\ & =\left(\frac{1}{2}\;\text{ln}\;2\pi +\;\text{ln}\;\sigma_{j}^{l}\right)\sum_{i=1}^{n}r_{ij}+\frac{1}{2} & \end{array}$$ (13)

This concludes the derivation of the information entropy in S2I-EM routing.

Since the value of information entropy is inversely proportional to the significance of Capsule features, it is a more reasonable choice to use $-S_{j}$ to measure the activation value of features. Ultimately, activation value *a*_*j*_ is compressed (interval 0-1) by the sigmoid function to more intuitively reflect the activation level of Capsule features.

$$\begin{array}{cc} & a_{j}=sigmoid\left(\gamma_{a}-\left(\gamma_{b}+\sum_{l=1}^{d}\;\text{ln}\;\sigma_{j}^{l}\right)\sum_{i=1}^{n}r_{ij}\right) \end{array}$$ (14)

where *γ*_*a*_, *γ*_*b*_ is a set of training parameters assigned to each upper-level Capsule, optimized by backpropagation.

4) ***Number of dynamic interests***. Let’s explore more details of the upper-level interest Capsule $Q_{j}$ calculation. The probability that behavior Capsule *i* is clustered into the same interest Capsule *j* is influenced by the similarity between voting matrices $V_{ij}$. Specifically, the voting matrix $V_{ij}$ is obtained by the transformation of behavioral Capsule *i*, i.e., behavior Capsule *i* and weight matrix $W_{ij}$ are multiplied by the product operation, where the weight matrix is learned by cost function and backpropagation.

$$\begin{array}{cc} & V_{ij}=P_{i}W_{ij} \end{array}$$ (15)

Note that the voting matrix $V_{ij}$ of each behavior Capsule *i* is associated with the predicted value of an element in interest Capsule *j*. That is, the mean value $\mu_{j}^{h}$ of the Gaussian distribution of elements in voting matrix indicates the h-th element in interest Capsule *j*. We essentially explain the process of S2I-EM routing execution, i.e., extracting multiple interest Capsules from the lower-level behavior Capsules to represent the UMI.

In addition, since user behaviors with implied interest diversity exhibit a distinct multi-peaked distribution, where the number of peaks corresponds to the number of interest Capsules. The value of K must be adjusted for different users. We employ heuristic rules to adjust how many interest Capsules will be given in the end. Specifically, the number of dynamic interests is determined by a combination of the Gaussian distribution of user behaviors and a predetermined K. The number of dynamic interests $K_{u}$ for user *u* is calculated as:

$$\begin{array}{cc} & K_{u}=max\left(1,min\left(K,{\text{log}}_{2}\left|I_{u}\right|\right)\right) \end{array}$$ (16)

We do not propose that all users employ the same value of K, because this dynamic technique of altering the number of user interests is primarily designed to provide better assistance for those users with fewer interests, in the meaning while, saving computing and memory resources. Algorithm 2 lists the whole S2I EM routing process.

### 3.4 Capsule-aware module

With the multi-interest representation learning module, we obtain multiple interest Capsules from user’s behavioral embeddings to represent different interests. Then we ask: *How to measure the importance of these interest Capsules?* We attempt to answer this question in the following perspective:

A user’s final behavior will be influenced by a certain interest, i.e., there is a clear correlation between the user’s actual behavior and a specific interest Capsule. Therefore, we designed a module based on a dot product attention mechanism during the training process, called Capsule-aware, for easier selection of interest Capsules to guide the model training. In Capsule-aware module, the label item is the query and the interest Capsules are both keys and values, as shown in Fig 2. Specifically, we first calculate the compatibility between each interest Capsule and the label item embedding. Second, we selected an interest Capsule corresponding to the maximum compatibility and calculated its weighted values, where the weight of the interest Capsule is determined by the corresponding compatibility. Finally, the user representation vector of the label item is represented by the weighted values of the selected interest Capsule and used to guide the training of our model. It’s worth noting that one of the primary reasons we didn’t utilize the weighted sum of all interest Capsules as the user representation vector for the label item throughout the training phase was to avoid confounding UMI and compromising the user’s maximum expectation. The target interest Capsule is obtained by the following equation:

$$\begin{array}{cc} & {\vec{q}}_{usim}=f_{att}\left({\vec{q}}_{j}^{⊤}{\vec{v}}_{i}\right) \end{array}$$ (17)

where ${\vec{q}}_{j}\in Q$ denotes representation vector of user’s *j* − *th* interest Capsule, $j\in \{1,\ldots ,K_{u}\}$. *f*_*att*_ is used to compute the similarity between labeled items and the interest Capsule. Thus, the final expression ${\vec{m}}_{u}$ for user *u* is computed as follows:

$$\begin{array}{cc} & {\vec{m}}_{u}=f_{concat}\left({\vec{q}}_{usim},{\vec{m}}_{u}\right) \end{array}$$ (18)

where *f*_*concat*_ represents the splicing function of user vector and the interest Capsule.

**Algorithm 2**: *S*2*I*-EM Routing

**Input**: user behavior embeddings $\left\{{\vec{v}}_{i},i\in I_{u}\right\}$, iteration times ***r***, number of interest Capsules K

**Output**: Return **Activations** ***a*_*j*_** and **Representation** $Q_{j},j=1,\ldots ,K_{u}$ of interest Capsules

**1** **Pre-processing**

**2** Initialize activations ***a*_*i*_ = 0.65** of behavior Capsules

**3** Calculate adaptive number of interest Capsules $K_{u}$ by (16)

**4** Calculate votes matrix $V_{ij}$: $V_{ij}={\vec{v}}_{i}W_{ij}$

**5** **Procedure *K*-*means***

**6** Arbitrarily choose $K_{u}$ objects from $I_{u}$ as the initial centroid, i.e., $C\leftarrow \text{RandomSimple}(I_{u},K_{u})$

**7** **repeat**

**8**  Assign each object ${\vec{v}}_{i}$ to its closest centroid

**9**  Update the cluster means, i.e., compute the new centroid (mean) of each cluster

**10** **until** *no change*;

**11** **return** $C=\{c_{1},c_{2},\ldots ,c_{j}\}$ is a set of ***k*** *cluster centroids*

**12** **Procedure *S*2*I*-*EM* ROUTING**

**13** $\forall i\in I_{u},j\in K_{u}:\text{Initialize}R_{\text{ij}}=1/\left|K_{u}\right|$

**14** **for** *iter = 1,…,r* **do**

**15**  $\forall j\in K_{u}:\text{M-STEP}\left(a,R,Q,S\right)$

**16**  $\forall i\in I_{u}:\text{E-STEP}\left(a,V,R\right)$

**17** **end**

**18** **return** a,Q

**19** **Procedure** M-STEP(a,Q,S)

**20** for all interest capsule *j*: $Q_{j}\leftarrow \sum_{i=1}r_{ij}V_{ij}$

**21** for all interest capsule *j*: $\sigma_{j}^{2}\leftarrow \sum_{i=1}r_{ij}{\left(V_{ij}-Q_{j}\right)}^{2}$

**22** for all interest capsule *j*: $S_{j}\leftarrow \left(\gamma_{b}+\sum_{l=1}^{d}\;\text{ln}\;\sigma_{j}^{l}\right)\sum_{i}r_{ij}$

**23** for all interest capsule *j*: $a_{j}\leftarrow \text{sigmoid}\left(\gamma_{a}-S_{j}\right)$

**24** **Procedure** E-STEP(a,V,R)

**25** for all behavior capsule *i*: $p_{ij}\leftarrow N\left(V_{ij};c_{j};\sigma_{j}^{2}\right)$

**26** for all behavior capsule *i*: $R_{ij}\leftarrow \frac{a_{j}p_{ij}}{\sum_{j=1}^{K_{u}}a_{j}p_{ij}},r_{ij}\leftarrow \frac{a_{i}R_{ij}}{\sum_{i}a_{i}R_{ij}}$

**27** **EndProcedure**

### 3.5 Multi-interest representation training objective

After the final user representation ${\vec{m}}_{u}$ and the label item embedding ${\vec{v}}_{i}$ are learned, we calculate the likelihood score *p*(*i*|*u*) with respective to candidate item *i* as:

$$\begin{array}{cc} & p\left(i|u\right)=p\left({\vec{v}}_{i}|{\vec{m}}_{u}\right)=\frac{exp({\vec{m}}_{u}^{⊤}{\vec{v}}_{i})}{\sum_{j=1}^{\left|I_{u}\right|}exp\left({\vec{m}}_{u}^{⊤}{\vec{v}}_{j}\right)} \end{array}$$ (19)

After that, we train MIRN by minimizing the probability of user *u* interacting with the target item *i*. In this sense, we can give the model a high level of flexibility and nonlinear modeling capabilities. The complete loss function of our MIRN is as follows:

$$\begin{array}{cc} & minL=-\sum_{u\in U,i\in (I-I_{u})}\text{log}\;p\left(i|u\right) \end{array}$$ (20)

The sum operator of Eq (19) is computationally expensive; thus, we use the sampled softmax technique [4] to make the objective function trackable and optimize the network by applying Adam [38], which is a variant of Stochastic Gradient Descent (SGD) to adapt the learning rate automatically. After training, the MIRN network except for the Capsule-aware module can be used as a user representation mapping function *f*_*user*_. And in the matching stage, the user’s behavior sequence and user base information are fed into the *f*_*user*_ function, generating multiple representation vectors for each user. These representative vectors are then used to retrieve the top N items through some approximate nearest neighbor methods, such as the Elasticsearch search engine [39]. Note that once the training is completed, we fix the model and construct approximate preference embeddings by fusing other attribute features of users to solve the cold start problem for new users.

## 4 Experiments

In experiments, we conduct extensive offline evaluations to verify that MIRN can improve both accuracy and diversity. In this section, we attempt to answer the following three research questions:

- RQ1: How does the proposed MIRN perform against different types of competitive models on recommendation accuracy in the matching stage?
- RQ2: What effect do different essential parameters have on MIRN’s accuracy and diversity?
- RQ3: How do the efficiency and complexity of MIRN compared to other models?

### 4.1 Offline evaluation

In this section, we present a comparison of the performance of MIRN and existing methods in the offline setting.

#### 4.1.1 Datasets and experimental setup

We evaluate recommendation performance on two publicly available datasets from different domains. One of them is the popular movie rating dataset MovieLens (https://grouplens.org/datasets/movielens/) provided by [40], which contains about 10 million user ratings from January 1996 to July 2014. The other is Amazon Product Data (http://jmcauley.ucsd.edu/data/amazon/) provided by [41], including user reviews (ratings, text, useful/unused votes, etc.) and product metadata (category, price, brand, etc.) on Amazon from May 1996 to July 2014. For MovieLens-Latest, due to the short user behavior sequence and simple data labels, we only consider the factors such as age, gender, occupation, and movie category. For Amazon Product Data, we deleted users whose operation records were less than 10 and items whose interaction records were less than 8. In addition, we consider the evaluation of fewer than three stars and above as positive, recorded as 1, and the evaluation of fewer than three stars as bad, recorded as 0. The statistics of the two datasets are shown in Table 2.

**Table 2:** Statistics of the two datasets for offline evaluation.

Dataset	Usrs	Categories	Items	Total
MovieLens-Latest	283,228	20	58,034	27,753,444
Amazon Electron	22,040	671	22,012	561,100
Amazon Movies-TV	35,832	15	28,545	752,676
Amazon Video-Games	5,459	59	4,258	83,748
Amazon Beauty	3,731	172	2,612	854,225
Amazon Office-Products	1,792	171	893	29,387
Amazon Home-Kitchen	11,511	67	7,727	143,088
Amazon Digit-Music	1,645	59	1,539	28,852

#### 4.1.2 Compared methods

- WALS [42] WALS, abbreviated as Weighted Alternating Least Squares, is a classical matrix decomposition algorithm. It performs the corresponding recommendation by confidence weights, giving a larger weight to items with high user preference and a smaller weight to items with no feedback.
- YouTube DNN [4]. In YouTube’s recommendation process, the division of the recommendation process into two phases, retrieval and sorting, is the most successful example in the field of recommendation systems.
- MaxMF [43]. It is a non-linear model. It uses multiple vectors to represent users, and then a vector to represent products. Finally, the largest score in the user vector is taken as the matching score.
- MIND [44]. The vectorized retrieval approach, represented by MIND, is one of the mainstream recommendation algorithms today. It generates multiple embeddings characterizing users’ interests to enhance the matching stage and avoids the problem of causing head effects.

#### 4.1.3 Evaluation metrics

We use the idea of sequential recommendation for prediction, i.e., we predict the next interaction between a user and an item, and thus evaluate the performance of the above method accordingly. We randomly divided the user-item interaction data into training set and test set by a ratio of 17:3. For each user, we first use the last interaction data as the target item and then use the remaining interaction data for that user as the user behavior.

- **Hit rate**. Hit rate (HR) is a commonly used metric for measuring recall in current top N recommendation studies. It calculates the percentage of underlying true items in the top N positions of the correctly ranked list, and a larger metric indicates better recommendation performance. The HR@N score is defined as:
  $$\begin{array}{cc} & HitRate@N=\frac{\sum_{\left(u,i\right)\in D_{test}}f_{i}\left(i_{target}\in T_{n}\right)}{\left|D_{test}\right|} \end{array}$$ (21)
  where $D_{test}$ denotes the test set consisting a large number of users and target items (*u*, *i*). $T_{n}$ is a set of the top N items in item ranking list and *f*_*i*_(⋅) is an indicator function.
- **Normalized discounted cumulative gain**. NDCG is a ranking performance evaluation metric widely used in recommender systems, which indirectly evaluates the performance of model by measuring the relevance of retrieved Top N item list to user *u*. The higher the relevance, the better the performance. So, it is defined as:
  $$\begin{array}{cc} & NDCG@N=\frac{1}{\left|U\right|}\sum_{u\in D_{test}}\frac{Rank\left(sim\right):\sum_{i=1}^{N}sim_{u}^{i}/\text{log}\;2\left(i+1\right)}{Rank\left(label\right):\sum_{i=1}^{N}sim_{u}^{i}/\text{log}\;2\left(i+1\right)} \end{array}$$ (22)
  where *Rank*(⋅) represents a ranking function on input sequence or values. $sim_{u}^{i}$ denotes the similarity score between the representation vector of user *u* and the embedding vector of candidate item *i*, and label is the true rating value of the item.

#### 4.1.4 Implementation details

In our experiments, we set the size of the embedding vector to 1024. We set the mini-batch size to 128 and use the Adam optimizer [38] with a 0.001 learning rate to minimize the total loss. In addition, we dynamically adjust the dropout rate in the range of {0.2, 0.4, 0.7} to prevent the overfitting of our models. And for other parameters, we use a grid search strategy to find the optimal settings. Finally, we adjust the *l*2 regularization term λ from 0.0001, increasing by a factor of 10 each time, and the learning rate from {0.001, 0.005, 0.01}. The implementation of the experiments is based on the server with the open-source framework Tensorflow-gpu2.4.0 [45], keras2.4.3 and python3.7, CUDA11.2. The GPU used for the experiments is NVIDIA GeForce RTX 3090 with 128GB memory.

#### 4.1.5 Model complexity analysis

In this subsection, we investigate the complexity of the proposed MIRN. It is obvious that the calculation of dynamic interest capsules and S2I-EM routing are the main operations. Given |*u*| users and |I| items, suppose that each user directly connects with $|I_{u}|$ items. In the process of calculating dynamic interest capsules, the computational complexity of procedure K-means is $O(K_{u})$, where $K_{u}$ denotes the number of interest capsules. In the process of S2I-EM routing, the time cost of the E-Step is $O(r|I_{u}|)$, and the M-Step requires time complexity $O(r|K_{u}|)$, where |*r*| represents the iteration times. Since the number of interest capsules $|K_{u}|$ is smaller than the number of items $|I_{u}|$ with which users interacted, we can regard it as a constant. The total time complexity is approximately $O(r|I_{u}|)$. Under the same experimental settings (i.e., representation sizes and initialized clusters), MIRN has a smaller complexity than MIND. Therefore, the total time complexity is acceptable in practice.

#### 4.1.6 Results analysis (RQ1)

We begin with the comparison w.r.t. HR@50 and NDCG@50. The empirical results are reported in Table 3, where %Imp. denotes the relative improvements of the best performing method (starred) over the strongest baselines (underlined). We find that:

**Table 3:** Performance comparison of different models on publicly available datasets.

	MovieLens Latest		Amazon Electron		Amazon Video-Games		Amazon Movies-TV	
Metrics@50	HR	NDCG	HR	NDCG	HR	NDCG	HR	NDCG
WALS	8.18	2.16	5.53	1.44	4.71	1.38	4.36	1.18
YouTube DNN	8.23	2.36	7.46	2.31	7.41	2.26	7.47	2.14
MaxMF	9.03	2.44	8.62	2.85	9.78	2.93	8.15	2.61
MIND	10.32	3.07	10.41	3.09	**10.22**	**3.11**	10.72	3.19
MIRN	**10.48**	**3.11**	**10.91**	**3.19**	10.19	3.01	**10.94**	**3.25**
Imp.	+1.55%	+1.30%	+4.80%	+3.23%	-0.29%	-3.32%	+2.05%	+1.88%

The best results are highlighted in bold. Numbers in the table indicate percentages, omitting the ‘%’.

- MIRN consistently outperforms most baselines across four datasets in terms of all measures. Specifically, it achieves significant improvements over the strongest baselines w.r.t. HR@50 by 4.80%, 2.05%, 1.55% in Amazon-Electron, Amazon Movie-TV, and MovieLens-Latest, respectively. This demonstrates the rationality and effectiveness of MIRN. We attribute these improvements to the multi-interest extraction capability of MIRN: (1) By mining users’ different interests, MIRN is able to better describe the relationships between users and items, and produce more robust representations of users and items. In contrast, other baselines ignore the importance of multiple interest representations, and use a single vector to model UMI; (2) Benefiting from our S2I-EM routing algorithm, MIRN can preserve the overall semantics of interest propensity and collect more information from behavioral Capsules, than USI-based baselines (i.e., WALS, YouTube DNN).
- Combining four datasets for MIRN analysis, we find that the improvement is more significant on Amazon-Electron than on Amazon-Video-Games. This is reasonable since (1) the user-item interaction data on Amazon-Electron provides denser and richer information than the data on Amazon-Video-Games; and (2) sparse interest categories dominate on Amazon-Video-Games. This suggests that MIRN is good at exploiting the potential of multi-interest extraction.
- Our MIRN achieves the best performance on several datasets, including Amazon Electron and Amazon Movies-TV. Compared to MIND the improvement is around 1% to 4%. For Amazon datasets, the improvements introduced by mining UMI are more significant, suggesting that users in Amazon tend to have more diverse interests in filtering items than in MovieLens-Latest.
- The matrix decomposition method, WALS, is defeated by other methods, indicating that deep learning is effective in improving the retrieval performance of the model. However, MaxMF performs much better than WALS without deep learning, which can be explained by the fact that MaxMF is a nonlinear model that uses multiple user representation vectors for retrieval. And we can also see from the results that the method using multiple user representation vectors (MaxMF, MIND) shows better performance than other methods (WALS, YouTube DNN). Thus, using multiple user representation vectors can effectively model UMI and boost the accuracy of recommendations.

In addition, we analyzed the experiments in which MIRN performed poorly by evaluating HR@10 and HR@50 in Table 4. An obvious finding is that these datasets (i.e., Amazon Office-Products, Amazon Beauty, and Amazon Digit-Music) are generally small (see Table 2 for details), making it difficult for the model to fit the data completely. While MaxFM performs better than MIRN on these datasets, we conjecture that (1) MaxFM benefits from the advantages of matrix factorization and can produce appropriate recommendations even with sparse data.; (2) It alleviates the high-dimensional disaster by introducing hidden vectors to decompose the high-dimensional behavioral sequences into multiple different representation vectors. This also demonstrates the effectiveness of using multiple Capsule vectors to represent UMI.

**Table 4:** Performance comparison of different models on other Amazon datasets.

	Amazon Office-Products		Amazon Home-Kitchen		Amazon Beauty		Amazon Digit-Music	
Metrics@10/50	HR@10	HR@50	HR@10	HR@50	HR@10	HR@50	HR@10	HR@50
WALS	0.0166	0.0635	0.0133	0.0482	0.0163	0.0617	0.0156	0.0621
YouTube DNN	0.0285	0.0762	0.0223	0.0750	0.0263	0.0758	0.0268	0.0757
MaxMF	**0.0291**	**0.0932**	0.0274	0.0861	**0.0284**	0.0934	0.0274	**0.0948**
MIND	0.0288	0.0926	0.0312	0.1045	0.0271	**0.0942**	**0.0278**	0.0922
Ours	0.0282	0.0921	**0.0314**	**0.1048**	0.0268	0.0916	0.0271	0.0931

The best results are highlighted in bold.

**Fig 3:**
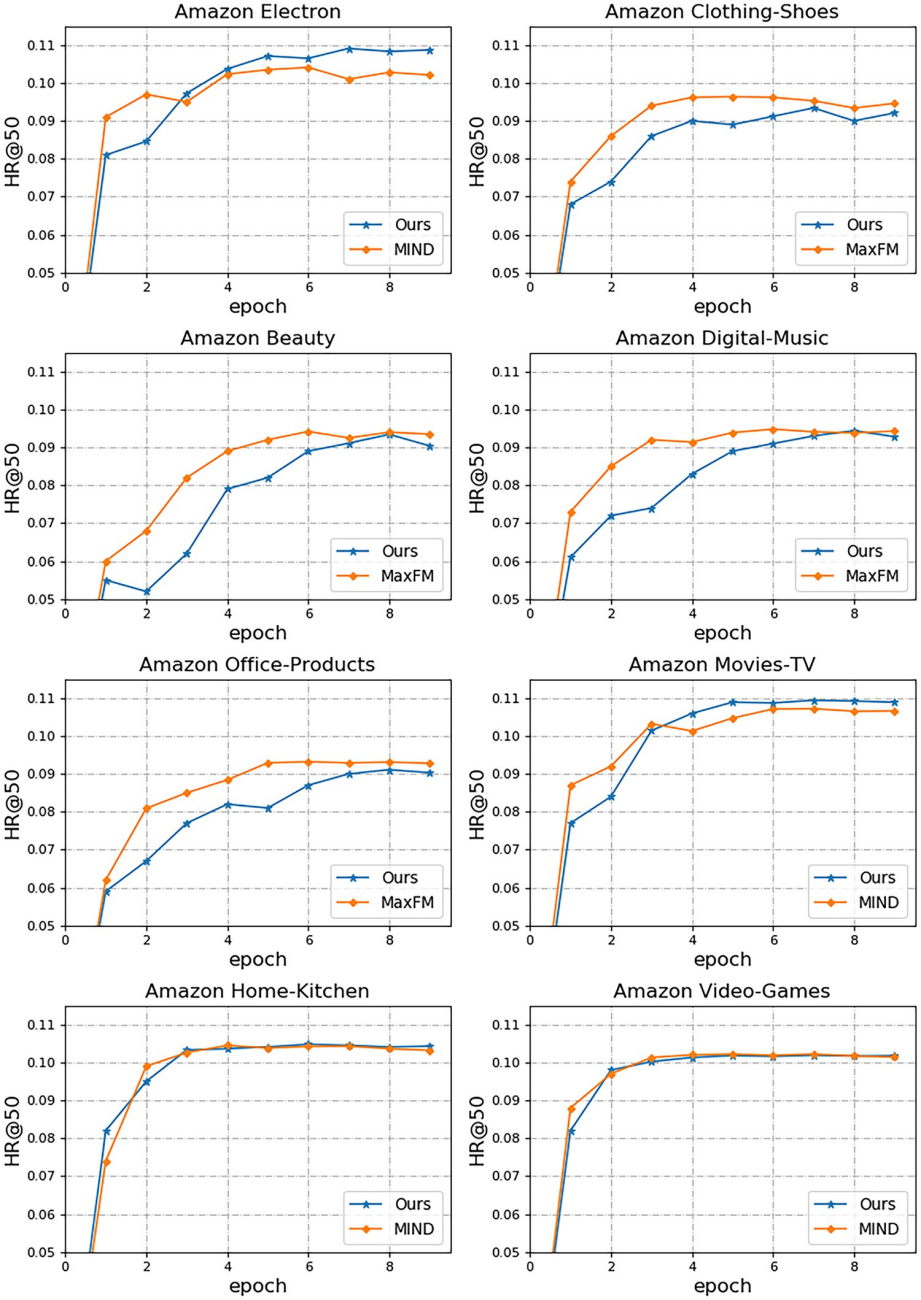
Comparison of HR@50 performance between our model and other models on different datasets.

The visualization results of the performance comparison of different models on different datasets are shown in Fig 3. From the experimental process of Amazon Electron with the Amazon Movies-TV dataset, it can be found that although MIRN requires longer iteration rounds compared to MIND, the performance achieved by MIRN is more advantageous. Besides, we found that there may be oscillations in our model-fitting curve when the data is small, which indicates that our model needs further optimization to learn enough relevant features when dealing with small datasets. However, the information collected by commercial platforms servicing millions of users is generally in the tens of thousands, so MIRN’s recommendation is more in accordance with the needs in real scenarios. Finally, we fine-tune our experiments on the Amazon Home-Kitchen and Amazon Video-Games datasets directly using the model already trained on Amazon Movies-TV. The model fitting curves show that our model learns the common behavioral patterns of users with good portability.

### 4.2 Discussion of hyperparameters (RQ2)

In this section, we conduct experiments on the Amazon Electron and Movielens-Latest datasets to investigate the effect of hyperparameters within the multi-interest representation learning module and Capsule-aware module, respectively.

1) ***Parameter K sensitivity***. We performed a corresponding sensitivity study on the hyperparameter K in the model architecture. K controls the overall capacity of the model. If K is small, the number of clusters is small for all the items; if K is big, the time complexity of the training and matching stage grows linearly with K. Moreover, a large K may increase the possibility of overfitting. Table 5 summarizes the performance of the model when the hyperparameter K is changed. And we can understand that the performance of our model steadily increases as K increases, peaking at K=5. For the Movielens-Latest dataset, the best performance of MIRN is obtained when K=5. For the Amazon Electron dataset, strong performance is still achieved when K=5.

**Table 5:** Model performance for different parameters K.

	Movielens-Latest				Amazon Electron			
	Metrics@10		Metrics@50		Metrics@10		Metrics@50	
Metrics	HR	NDCG	HR	NDCG	HR	NDCG	HR	NDCG
MIND(K=3)	31.17	6.83	**48.46**	**15.34**	3.09	1.18	10.41	1.47
MIRN(K=1)	30.24	5.60	48.31	10.66	2.21	0.82	9.57	1.03
MIRN(K=3)	31.77	6.25	48.34	12.28	**3.19**	**1.01**	10.02	1.36
MIRN(K=5)	**32.71**	**7.47**	48.40	13.53	3.16	0.97	**10.91**	**1.88**
MIRN(K=7)	31.15	7.06	48.32	13.01	3.14	0.81	10.71	1.56

All figures are presented as percentages and “%” is omitted.

**Fig 4:**
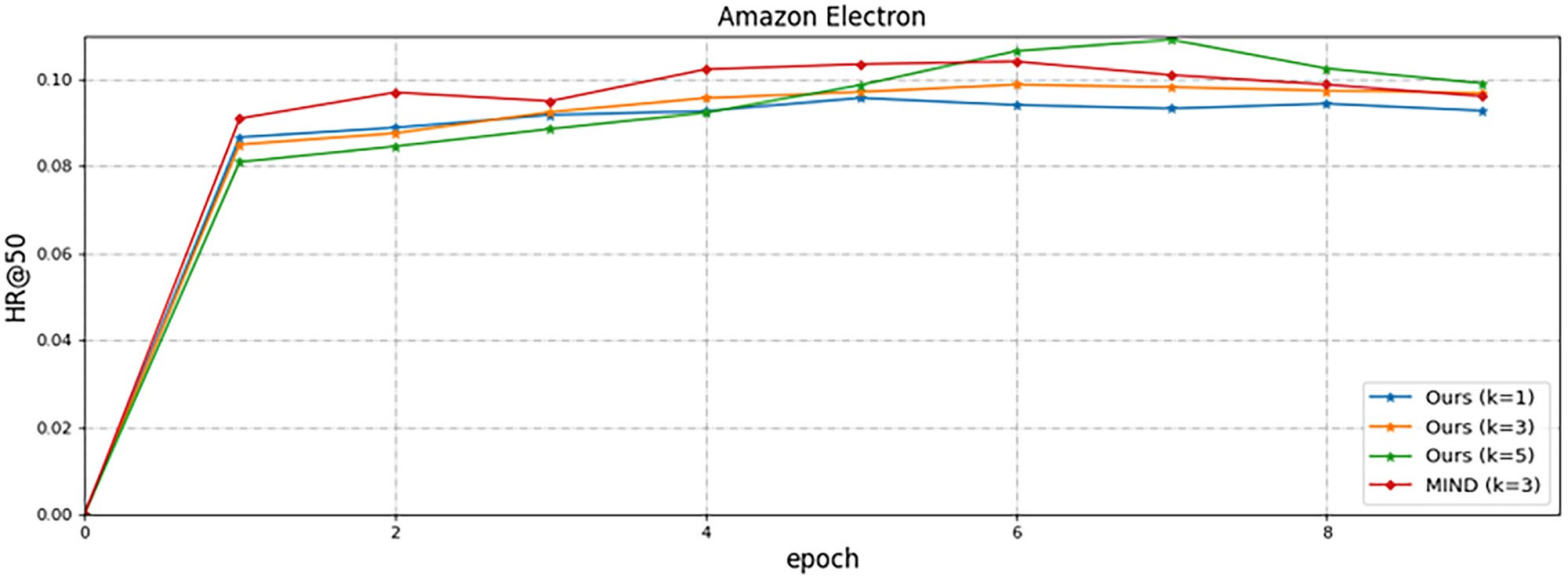
Performance comparison of different numbers of interest K in MIRN. The y-axis represents the value of HR@50 and the x-axis gives the number of iterations. MIRN performs better with bigger K.

Fig 4 shows the iterative fitting speed and accuracy of MIRN on the Amazon Electron dataset. We notice that the performance is worst when K=1. This is because users show the same intention for all items, making the accuracy of the model suboptimal due to the lack of interest diversity. However, as K rises, user interest diversity gradually surfaces, user-side representations get richer, and the model is able to capture more accurate information to improve the ability of item retrieval. Compared to MIND, our model exhibits higher performance with different K values, which demonstrates the efficacy of our modeling strategy for extracting multiple interests.

2) ***Connection probability***. The connection probability *R*_*ij*_ is used to quantify the correlation between the behavior Capsules and the interest Capsules. The value of the connection probability fluctuates continually over the algorithm’s iterations, and in this study, we visualize its value to show that the multi-interest extraction process is interpretable.

**Fig 5:**
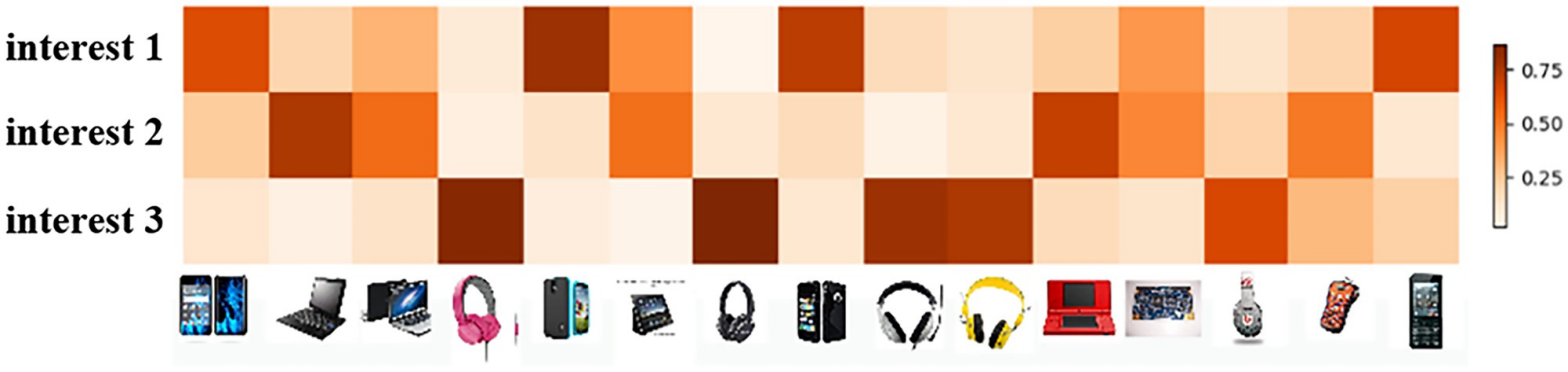
Heatmap of coupling coefficients for user behaviors. Each behavior has a corresponding coupling coefficient in the corresponding capsule of interest.

Fig 5 illustrates the connection probabilities associated with a user, where each row represents one of the user’s interest Capsules, and each column corresponds to one of the user’s behaviors. The intensity of the color in heat map indicates the user’s preference for the item. A dark color indicates a strong preference for the item and, conversely, a light color implies a lack of interest. From the results, we can see that the user interacts most frequently with three categories of goods (cell phones, computers, and headphones), and their respective connection probabilities are the largest in the interest Capsule, i.e., these three categories of goods form the user’s corresponding interest. Regarding this result, we believe that the work of extracting multiple interests of the user has been achieved, i.e., the UMI are divided into different interest Capsules.

### 4.3 Model efficiency and complexity (RQ3)

We analyze the efficiency and complexity of retrieval models in various terms. In Table 6, we report the number of parameters, the number of floating-point operations (FLOPs), and the inference latency of item retrieval in the matching stage. MaxFM learns nonlinear latent factors to represent multiple user vectors, leading to a disadvantage in parametric number and inference time. YouTube DNN represents a user with a long single vector, which has fewer parameters and FLOPs than MaxFM, but its accuracy is likewise low. This demonstrates that the use of multiple vectors to represent the user can make a greater contribution to improving models’ accuracy. In addition, some of the more advanced models, such as MIND and MIRN, take the form of multi-vector representations, as indicated in Table 6. It can be observed that MIRN with a composite clustering strategy not only outperforms its much larger counterpart, MIND, in terms of performance; its reduced model size also significantly reduces inference costs and greatly facilitates the use of smaller hardware in downstream tasks. Second, in our analysis, the number of initialized interest categories K and the length of user behavior sequences are the key factors affecting FLOPs. Increasing the value of K increases the number of parameters in our model marginally while also improving its performance. In conclusion, MIRN outperforms other methods in terms of model size and multi-interest extraction.

**Table 6:** Comparison of the speed and accuracy of different dense retrieval models on Amazon Electron.

Models	Params. (M)	FLOPs (G)	Latency (ms)	HR@50 (%)
Youtube DNN	93	16.4	61.2	7.46
MaxFM	147	19.6	73.7	8.62
MIND(K=3)	67	14.1	47.9	10.41
MIRN(K=3)	54	13.3	46.4	10.02
MIRN(K=5)	61	13.8	47.3	10.91

The latency in this table is measured without post-processing. Latency is counted on a workstation with Intel E5-2650 v2 CPU and NVIDIA GeForce RTX 3090 GPU.

## 5 Conclusion and future works

In this paper, we propose a new model named MIRN, which can generate multiple Capsule vectors of users’ different interest representations for item retrieval in the matching stage. Specifically, we design an S2I EM routing algorithm to dynamically extract the different interests of users, and then these interests are trained with the Capsule-aware module. Besides, a composite clustering strategy is applied in this paper to make our model more lightweight. To investigate the performance of MIRN, we conducted extensive offline evaluations, results analysis. Experiments have shown that our model achieves significant improvements compared to the baseline approaches on two large public datasets, Movielens-Latest and Amazon Product. In the future, we will work in two directions. The first is to build a multi-channel network to capture the temporary interests generated by users in a short time. The second direction is to investigate the embedding of user preference with the GNN and metric-based learning techniques in order to enhance the performance of cold-start recommendation.
